# Impact of Bt corn expressing *Bacillus thuringiensis* Berliner insecticidal proteins on the growth and survival of *Spodoptera frugiperda* larvae in Colombia

**DOI:** 10.3389/finsc.2024.1268092

**Published:** 2024-02-23

**Authors:** Jairo Rodriguez-Chalarca, Sandra J. Valencia, Alejandra Rivas-Cano, Francisco Santos-González, Diana Patricia Romero

**Affiliations:** ^1^ Crops for Nutrition and Health, The Alliance of Bioversity International and Centro Internacional de Agricultura Tropical (International Center for Tropical Agriculture), Palmira, Colombia; ^2^ Universidad Nacional de Colombia Sede Palmira, Palmira, Colombia; ^3^ Resistance Management NorLa-Bayer, Mexico City, Mexico; ^4^ Bayer – Regulatory, Bogotá, Colombia

**Keywords:** Bt corn, Cry1a.105, Cry2Ab2, *Spodoptera frugiperda*, growth inhibition

## Abstract

Bioassays were conducted under controlled conditions to determine the response of *Spodoptera frugiperda* (J. E. Smith) larvae fed with corn materials expressing *Bacillus thuringiensis* (Bt) insecticidal endotoxins: (1) VT Double Pro^®^ (VT2P) expressing Cry1A.105-Cry2Ab2 proteins and (2) VT Triple Pro^®^ (VT3P) expressing Cry1A.105-Cry2Ab2-Cry3Bb1 proteins. The parameters assessed were: (i) mortality rate, and (ii) growth inhibition (GI) with respect to the control. To conduct this study, larvae were collected from commercial non-Bt corn fields, in four agricultural sub-regions in Colombia, between 2018 and 2020. Fifty-two populations were assessed from the field and neonate larvae from each of the populations were used for the bioassays. The study found that mortality rates in the regions for larvae fed with VT2P corn ranged from 95.1 to 100.0%, with a growth inhibition (%GI) higher than 76.0%. Similarly, mortality rate for larvae fed with VT3P corn were between 91.4 and 100.0%, with a %GI above 74.0%. The population collected in Agua Blanca (Espinal, Tolima; Colombia) in 2020, showed the lowest mortality rate of 53.2% and a %GI of 73.5%, with respect to the control. The population that exhibited the lowest %GI was collected in 2018 in Agua Blanca (Espinal, Tolima, Colombia) with a 30.2%, growth inhibition, with respect to the control. In recent years, the use of plant tissue to monitor susceptibility to fall armyworm has proven to be useful in the resistance management program for corn in Colombia determining that the FAW populations are still susceptible to Bt proteins contained in VT2P and VT3P.

## Introduction

Fall armyworm, *Spodoptera frugiperda*, is one of the most serious pests in agricultural production in the Americas (1, 2). It is distributed widely among hosts in the Western Hemisphere (in more than 27 plant families), it can migrate very easily (3–6), and has had a big economic impact on corn, sorghum, rice, and cotton crops (2, 3, 7, 8). *S. frugiperda* was first reported in West Africa in 2016 (2, 9, 10) and then spread throughout much of sub-Saharan Africa by 2017 (11). In 2018, it was reported in India and China, where it probably migrated from Africa (5, 12, 13).

The inclusion of Bt crops in agricultural systems has brought enormous economic and environmental benefits, such as higher yields, due to less damage to plants, and a significant reduction in the use of insecticides (14, 15). Special academic interest has been shown in strategies that prevent or delay the emergence of resistance by target insects to Bt (16–19).

Although most target populations still show high susceptibility to Bt crops, under field conditions resistance in biotech corn and cotton has been documented for some lepidopteran pests which include: *Busseola fusca* in South Africa to Cry1Ab protein (20, 21); *S. frugiperda* in Puerto Rico to Cry1F protein (22); *Pectinophora gossypiella*, in western India to Cry1Ac; *Helicoverpa zea* in the southwestern United States (Cry1Ac and Cry2Ab2), and for *Helicoverpa punctigera* in Australia to (Cry1Ac and Cry2Ab) (21, 23–25). The reported emergence of resistance in the field was demonstrated by evidence of increased damage by the target insect, that is to say, the loss of efficacy of the biotech crop to control *B. fusca*, *S. frugiperda, P. gossypiella* and *H. zea* (20–22, 24–26). The widespread use of Bt crops offers an opportunity to test the hypothesis of how responses to selection pressure are influenced by various genetic and ecological factors (21).

The main purpose of monitoring the susceptibility of target pests in Bt crops, is to be able to detect changes in the response to the action of proteins that allow corrective actions to be taken before the control measure loses its efficacy (27, 28). Therefore, as mentioned above, changes in the susceptibility of target pests based on the LD_50_ variation do not necessarily imply changes in susceptibility under field conditions (26, 29). This means that regulatory decisions on the continued use of Bt technologies must include information about the relationship between variation in susceptibility and control under field conditions (24).

In Colombia, *S. frugiperda* is the most damaging pest in corn production. It was first reported in 1917 in the department of Antioquia. It then appeared in Caldas and Valle del Cauca, and then spread to all corn growing areas (30). Losses of up to 60% have been reported in Colombia due to *S. frugiperda*, which can increase production costs from 5.6 to 10.0% for technified corn (31–33). Blanco et al. (8), reported *S. frugiperda* to be the most damaging pest for non-Bt corn production in Colombia. Using chemicals to control *S. frugiperda* (organophosphates and pyrethroids) has become the most widely used method of reducing the impact of the insect in the field (33). These control measures have had a significant impact on the environment and the ability to resist *S. frugiperda*. This has prompted the need to look for new alternatives, such as the use of pathogens as control agents (34, 35). *B. thuringiensis* Berliner is one of the biggest species with insecticidal capacity (36) and its results are similar to chemical control (34, 37). Additionally, the adoption of genetically modified crops (such as Bt corn that express Cry and Vip toxins derived from bacterium *B. thuringiensis* var. *kurstaki)*, have become another alternative for reducing the impact of *S. frugiperda* (35). Bt crops are an important tool for the control of *S. frugiperda* (38), that significantly reduce the need for insecticides and ensure protection of the crop throughout its growth (39, 40).

In Colombia, the introduction, release, and commercialization of GM crops is carried out under the Cartagena Protocol on Biosecurity, an international instrument that regulates living modified organisms (LMOs) which are the product of modern biotechnology. Colombia has been sowing genetically modified (GM) crops in its production systems since 2002. 2,000 ha of cotton was the first crop to be planted. In 2007, 6,000 ha of corn was then authorized and sown (41). The number of GM hectares sown in Colombia then increased significantly. By 2021, 31.6% more land had been sown with GM crops than the previous year. That is a total of 150,451 ha, of which 142,975 ha was GM corn, 7,464 GM cotton, and 12 ha blue flowers (cut) (42).

The purpose of this study is to assess the effect of the consumption of corn containing Bt proteins on the mortality and growth of *S. frugiperda* larvae collected under field conditions within the Insects Resistance Management (IRM) monitoring program in Colombia.

## Materials and methods

### Larvae collection and establishing colonies

Larvae were collected from 49 locations during the period 2018 to 2020, in commercial plots with conventional corn (non-Bt) from the four agricultural subregions of Colombia (**Table 1**). In each location, three hundred larvae in different stages of development (F0) were collected and taken to the CIAT (International Center for Tropical Agriculture) facilities, where each collection was handled separately, to obtain the F1 necessary for the assembly of the corresponding bioassays.

**Table 1:** *S. frugiperda* collections made between 2018 and 2020 on non-Bt corn.

Year	Region	Population	Municipality	Department	N	W
2018	HC	Carrillo	San Pelayo	Córdoba	8.77455	75.86673
2018	HC	El Tajo	Cereté	Córdoba	8.90480	75.80452
2018	HC	La Villa	San Benito de Abad	Sucre	8.56967	75.02471
2018	HC	Martínez	Cereté	Córdoba	8.88263	75.76979
2018	GVCR	Guayabita	Cartago	Valle	NA	NA
2018	GVMR	Aguablanca alta	Espinal	Tolima	4.18026	74.91654
2018	GVMR	Dindalito-centro	Espinal	Tolima	4.17384	74.94717
2018	GVMR	Serrezuela	Guamo	Tolima	4.07332	74.93216
2018	Orinoquia	El crucero	Granada	Meta	3.45993	73.78998
2018	Orinoquia	El Toro	Puerto López	Meta	4.19276	72.44249
2018	Orinoquia	La Cristalina	Puerto Gaitán	Meta	3.48071	73.72449
2019	HC	El playón	Lorica	Córdoba	9.18662	75.81989
2019	HC	La Ceibita	Cereté	Córdoba	8.85008	75.76283
2019	HC	Las Cachuchas	Cereté	Córdoba	8.82296	75.80196
2019	HC	Mocarí	Montería	Córdoba	8.811	76.8483
2019	HC	Parcelas de Venezuela	Tierra Alta	Córdoba	8.21972	76.00827
2019	HC	Playa Rica	Cereté	Córdoba	8.87616	75. 810426
2019	GVCR	Morelia	Roldanillo	Valle	4.46858	76.10598
2019	GVCR	ND	Palmira	Valle	3.525	76.286111
2019	GVMR	Dindalito	Espinal	Tolima	4.1692	74.8819
2019	GVMR	Montalvo	Espinal	Tolima	4.17088	74.86059
2019	GVMR	ND	Espinal	Tolima	4.127222	74.840083
2019	GVMR	Paradero	Flandes	Tolima	4.2323	74.8282
2019	GVMR	Samán	Espinal	Tolima	4.1709	74.9712
2019	GVMR	San Jorge	Guamo	Tolima	4.07433	74.93868
2019	GVMR	Talura	Espinal	Tolima	4.7.27	74.51.37
2019	Orinoquia	Crucero	Granada	Meta	3.46238	73.76679
2019	Orinoquia	El Toro	Puerto López	Meta	4.18926	72.44507
2019	Orinoquia	ND	Puerto López	Meta	4.245	72.4092
2020	HC	Buena Vista	Cereté	Córdoba	8.93300	75.74962
2020	HC	Cabuya	San Carlos	Córdoba	8.82333	75.71000
2020	HC	El Cedro	Cereté	Córdoba	8.74662	75.81405
2020	HC	Martínez	Cereté	Córdoba	8.87333	75.73550
2020	HC	Martínez	Cereté	Córdoba	8.88472	75.74667
2020	HC	ND	Cereté	Córdoba	8.90276	75.79983
2020	HC	San Antonio	Cereté	Córdoba	NA	NA
2020	GVCR	La legua	Yotoco	Valle	3.85389	76.36083
2020	GVCR	La selva	Ginebra	Valle	NA	NA
2020	GVCR	ND	Riofrio	Valle	4.10875	76.29695
2020	GVCR	Tierra Blanca	Roldanillo	Valle	4.42229	76.12904
2020	GVMR	Aguablanca alta	Espinal	Tolima	3.80386	73.30049
2020	GVMR	Buenos Aires	Ibagué	Tolima	4.19493	75.04212
2020	GVMR	Canastos	Espinal	Tolima	NA	NA
2020	GVMR	Cardonal	Espinal	Cardonal	4.11796	74.91613
2020	GVMR	Colegio	Flandes	Tolima	4.21991	74.83854
2020	GVMR	Rincón de San Fco.	Espinal	Tolima	4.19451	74.95269
2020	Orinoquia	Bajo Pompeya	Villavicencio	Meta	3.99036	73.31725
2020	Orinoquia	Surimena	San Carlos de Guaroa	Meta	3.80386	73.30049
2020	Orinoquia	Yurimera	Puerto López	Meta	NA	NA

HC, Humid Caribbean.

GVCR, Geographic Valley of the Cauca River.

GVMR, Geographic Valley of the Magdalena River.

NA, Not available.

GCS, Geographic Coordinate System.

Each collection (**Table 1**) represented an independent colony, which was kept under controlled conditions; 27 ± 2°C, 14:10 (light/dark) artificial photoperiod and 70-75% relative humidity. Adults were housed in cages (35 cm long, 25 cm wide and 25 cm high) and labeled according to where they were collected (**Table 1**). Adults were fed with a water solution and pasteurized honey (1:1 vol/vol) using absorbent wet cotton that was kept in small containers inside the cage. Egg masses were removed and deposited individually in labeled petri dishes until larvae emerged (F1). Neonate larvae were quarantined for 24 hours prior to setting up bioassays to correct natural mortality.

### Feeding bioassays with Bt material

Plant materials used included commercial hybrids SV-1035 (non-Bt) and two hybrids (Bt), VT Double Pro^®^ (VT2P) and VT Triple Pro^®^ (VT3P) (**Table 2**). About Bt materials, 32 replicates were assessed, each with 16 larval neonates (<24 hours) with a total of 512 larvae per plant material. For the non-Bt material (control), 8 replicates with 16 larvae each were used for a total of 128 larvae.

**Table 2:** Bt and non-Bt corn materials were assessed in this study.

Corn ID	Bt gene	tradename	Traits^1^	Event
Non-Bt	None	SV-1035	Non-Bt	None
VT2P	Cry1A.105 – Cry2Ab2	–	VT Double PRO^®^	MON 89034 X NK603
VT3P	Cry1A.105 – Cry2Ab2- Cry3Bb1	–	VT Triple PRO^®^	MON 89034 X MON 88017

^1^Commercial hybrids in Colombia

The materials assessed were sown under semi-controlled conditions in a screen house at the CIAT campus in Palmira, Valle del Cauca, Colombia. When the materials reached vegetative stage V6 - V8, the youngest fully expanded leaves were harvested and taken to the laboratory for bioassays (43). Before starting the bioassays, random samples were taken from the plants, which were analyzed to confirm the presence of the proteins using a strip test. Bioassay trays (White Rearing Tray 32 cells, ref: RT32W, Frontier Agriculture Sciences) were used for the bioassays.

Each cell was inoculated with one neonate larva (24 hours after emerging) and placed in an air-conditioned chamber at 27± 1° C, in 60% relative humidity in darkness for 7 days. Leaf portions (measuring 9.0 cm^2^) from each of the materials were used as food. To make sure the larvae food was fresh, it was replaced every 48 hours with freshly harvested material from the mesh house. A cotton ball moistened with distilled water was placed in each of the wells, to maintain adequate humidity (44).

The assessed parameters were: (i) mortality rate 7 days after consuming corn (dac) and (ii) growth inhibition (%GI) with respect to the larvae control that managed to survive 7dd (45–48).

With regard to the 7 days after consuming corn (dac) mortality record, it was found that larvae that had not passed the first stage and those that weighed ≤10 mg were considered dead (46, 49).

With reference to the control, growth inhibition (%GI) was calculated by dividing the average weight of surviving larvae in each Bt material (VT2P, VT3P) by the average weight of larvae in the control (non-Bt) and shown as a percentage. In each of the materials, live larvae were individually weighed to obtain the average using a Sartorius Quintix35 -1S precision balance (Sartorius Lab Instruments GmbH & Co) (50).

### Data analysis

Mortality was measured 7 days after consuming (dac) Bt corn plant material and understood as the sum of (dead larvae + Larvae L1 and/or weight ≤10 mg) (46). Mortality data were corrected with the Henderson and Tilton (H&T) formula (51). Tukey’s test (P<0.05) and GLM for multiple comparisons (52) were used to compare means.

## Results

**Figure 1:**
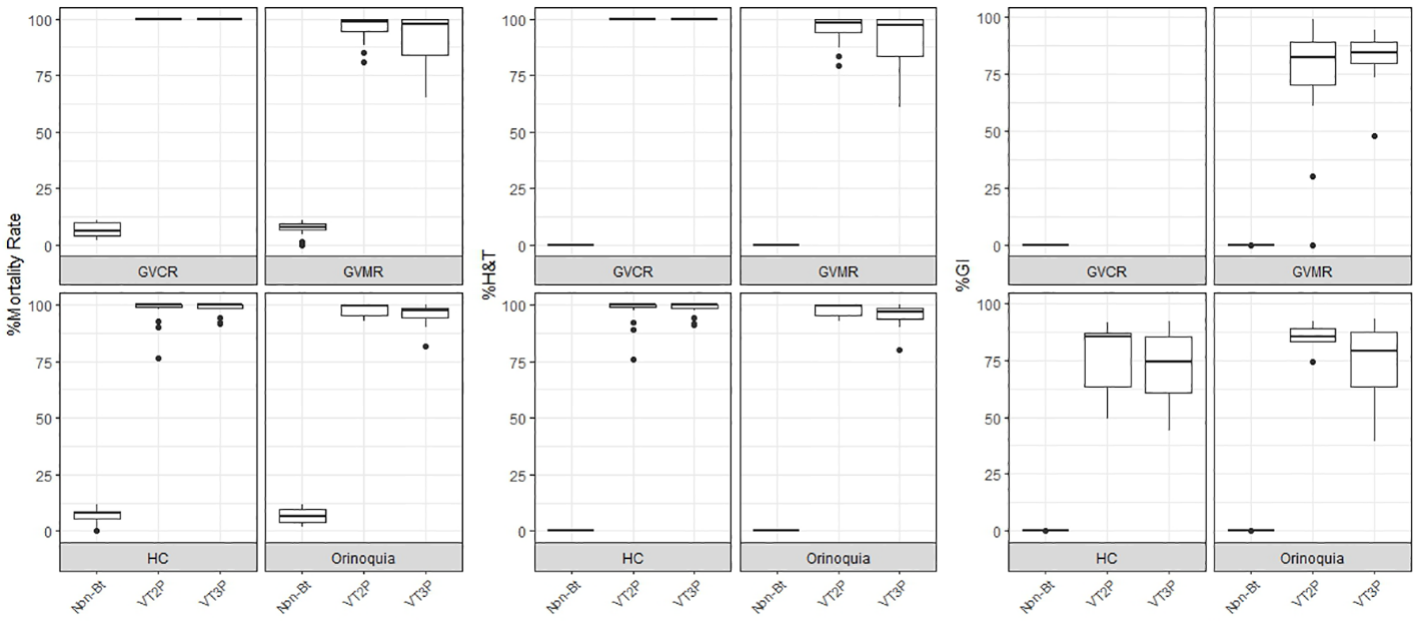
Response of *S. frugiperda* populations, according to % Mortality Rate, % H&T and Growth Inhibition (%GI), following the consumption of corn materials with VT2P and VT3P technology in Colombia, for collections 2018 to 2020.

This section summarizes the information on the response of 49 *S. frugiperda* populations to the effect of VT2P and VT3P corn consumption from Colombia’s four agricultural subregions between 2018 and 2020 (**Table 2**, **Figure 1**).

With respect to the interaction between corn materials (Non-Bt, VT2P, VT3P) and the year collected, there were significant differences in mortality rates, with values ranging from 8.6% after consuming non-Bt corn to 98.6% after consuming VT3P corn (**Table 3**). Growth inhibition (%GI) because of consuming Bt corn, showed statistical differences, with values ranging between 56.3 and 86.8% for VT2P (2018) and VT3P (2019), respectively (**Table 3**, **Figure 1**).

**Table 3:** Means ± SD of mortality rate and growth inhibition 7 days after consuming (dac) non-Bt, VT2P and VT3P corn for *S. frugiperda* populations collected between 2018 and 2020.

Corn ID	year	No.	Mortality rate (%)^1a^	H&T^2b^	%GI^3c^
Non-Bt	2018	1,408	7.9 ± 2.6 b	0.0	0.0
	2019	2,304	6.1 ± 3.6 b	0.0	0.0
	2020	2,560	7.3 ± 3.1 b	0.0	0.0
VT2P	2018	5,632	97.8 ± 3.9 a	97.6 a	56.3 ± 16.7 c
	2019	9,216	98.0 ± 5.6 a	98.0 a	84.5 ± 8.9 a
	2020	10,240	96.1 ± 5.6 a	95.8 a	77.0 ± 26.8 ab
VT3P	2018	5,632	96.7 ± 5.7 a	96.5 a	61.9 ± 13.4 bc
	2019	9,216	98.4 ± 2.4 a	98.2 a	86.6 ± 4.8 a
	2020	10,240	93.7 ± 9.4 a	93.1 a	78.3 ± 16.1 ab

n: Number of total larvae assessed each year.

SV-1035: conventional corn hybrid (expressing no Bt protein), control.

^1^Mortality: Dead larvae + L1 larvae and/or larvae weighing ≤10 mg) ± SD.

^2^Mortality rate: H&T corrected mortality.

^3^Percentage of weight loss for live larvae 7 dac, with respect to the control (non-Bt corn).

In each column, values followed by different letters are statistically different (P<0.05) according to Tukey’s test and GLM for multiple comparisons.

a ANOVA results: df=8; F=1,039.69; P<0.001.

b ANOVA results: df=8; F=1,256.88; P<0.001.

c ANOVA results: df=8; F=287.80; P<0.001.

**Figure 2:**
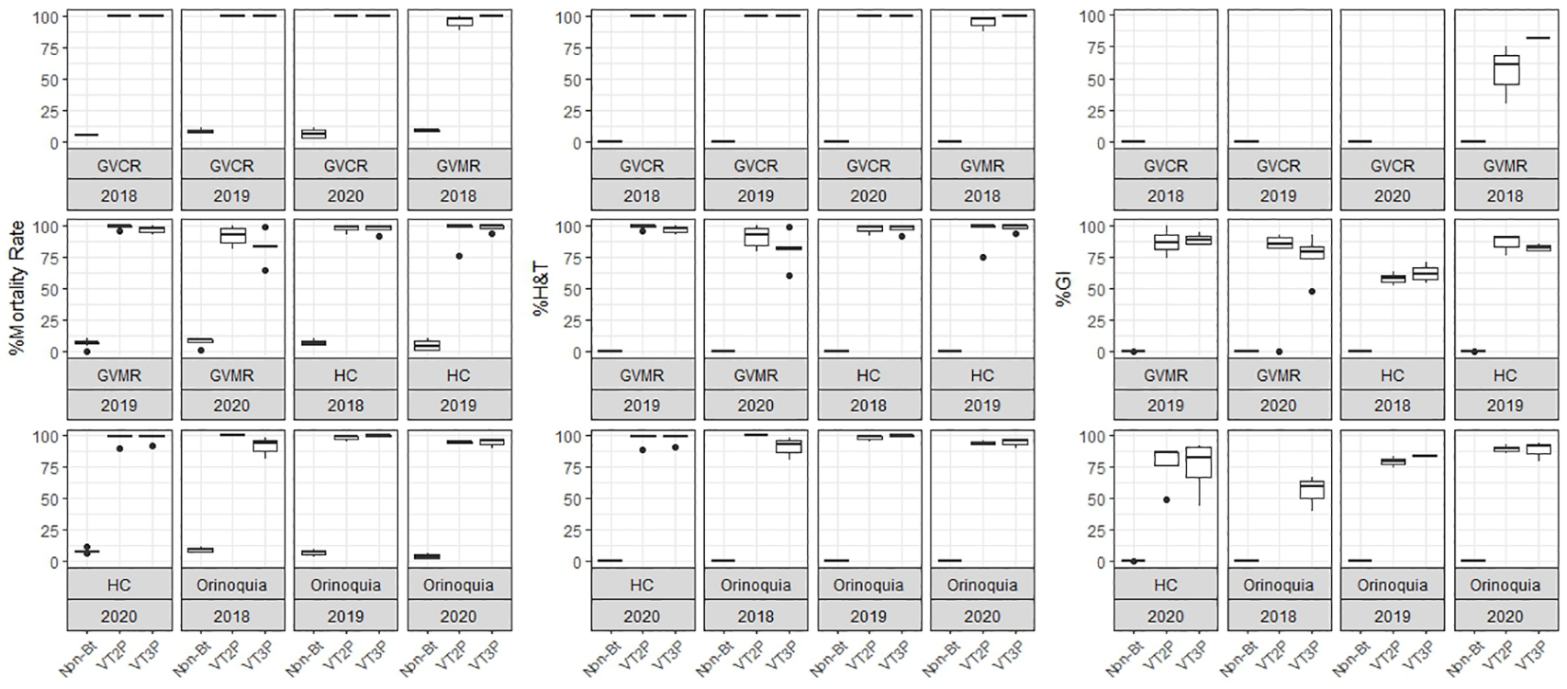
Response of *S. frugiperda* populations, according to % Mortality Rate, % H&T and Growth Inhibition (%GI), because of consuming maize materials with VT2P and VT3P technology for four agricultural subregions of Colombia for collections 2018 to 2020.

The response of *S. frugiperda* populations to the corn material collected from the four agricultural subregions showed statistical differences for mortality rates after consuming Non-Bt and Bt corn (**Table 4**). Values ranged from 7.1% for the Orinoco populations fed with non-Bt corn to 100.0% for those collected in GVCR and fed with VT2P and VT3P corn. With respect to growth inhibition (GI), there were extreme values in HC collections, with 74.2% and 84.5% recorded for collections from Orinoco, fed with VT3P and VT2P, respectively (**Table 4**, **Figure 2**).

**Table 4:** Means ± SD of mortality rate and growth inhibition 7 days after consuming (dac) non-Bt, VT2P and VT3P corn on *S. frugiperda* populations collected in the four agricultural subregions.

Region	Corn	No.	Mortality rate (%)^1a^	H&T^2b^	%GI^3c^
HC	Non-Bt	2,176	6.8 ± 3.4 c	0.0	0.0
HC	VT2P	8,704	97.3 ± 6.1 ab	97.1 b	75.7 ± 16.7 a
HC	VT3P	8,704	98.4 ± 2.8 ab	98.3 ab	72.2 ± 16.6 a
Orinoquia	Non-Bt	1,152	6.6 ± 3.3 c	0.0	0.0
Orinoquia	VT2P	4,608	97.6 ± 2.9 ab	97.5 ab	84.7 ± 6.8 a
Orinoquia	VT3P	4,608	95.2 ± 6.0 ab	94.9 ab	73.3 ± 19.3 a
GCVR	Non-Bt	896	6.9 ± 3.5 c	0.0	0.0
GCVR	VT2P	3,584	100.0 ± 0.0 a	100.0 a	–
GCVR	VT3P	3,584	100.0 ± 0.0 a	100.0 a	–
GMVR	Non-Bt	2,048	7.5 ± 3.1 c	0.0	0.0
GMVR	VT2P	8,192	95.7 ± 6.1 ab	95.3 ab	71.7 ± 29.0 a
GMVR	VT3P	8,192	92.4 ± 9.9 b	91.7 b	81.8 ± 12.3 a

n: Number of total larvae assessed each year.

SV-1035: conventional corn hybrid (expressing no Bt protein), control.

^1^Mortality: dead larvae + larvae and/or larvae weighing ≤10 mg), ± STDEV.

^2^Mortality rate: H&T corrected mortality.

^3^Percentage of growth inhibition for live larvae growth 7 dac, with respect to the control (non-Bt corn).

In each column, values followed by different letters are statistically different (P<0.05) according to Tukey’s test and GLM for multiple comparisons.

a ANOVA results: df=11; F=781.04; P<0.001.

b ANOVA results: df=11; F=962.29; P<0.001.

c ANOVA results: df=9; F=138.51; P<0.001.

### Susceptibility of humid Caribbean populations

**Figure 3:**
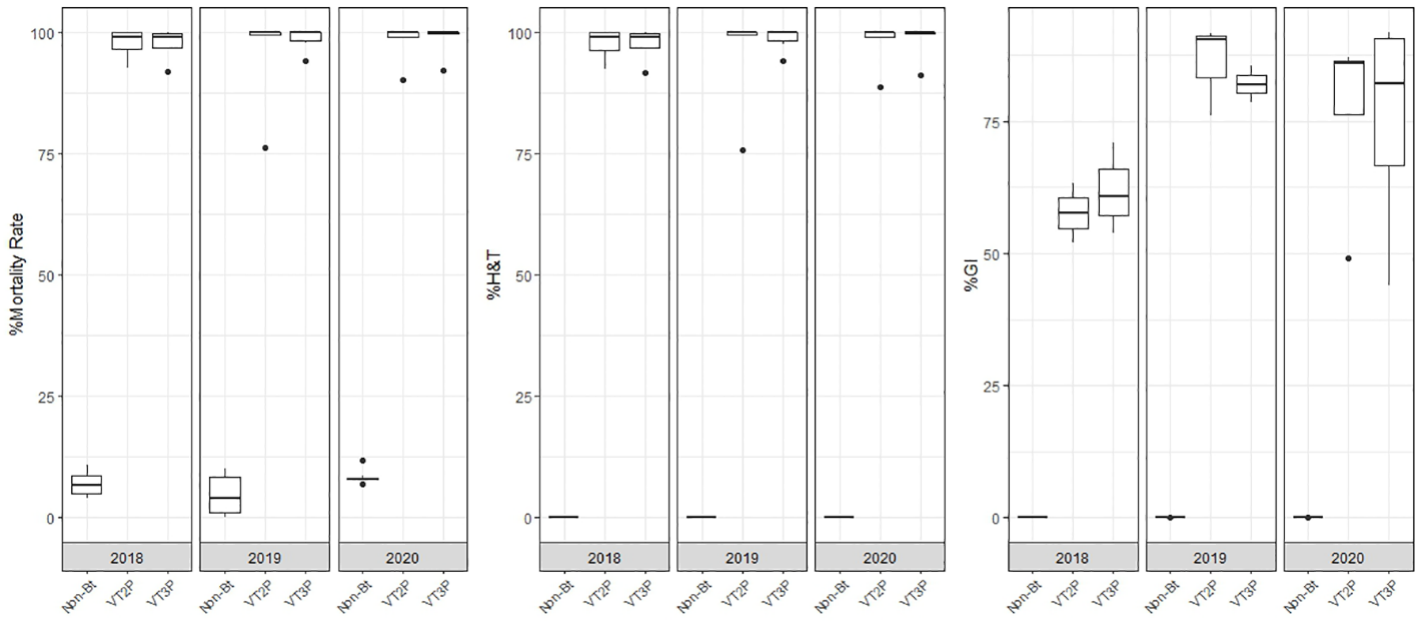
Response of *S. frugiperda* according to according to % Mortality Rate, % H&T and Growth Inhibition (%GI), due to consuming corn materials with VT2P and VT3P technology in HC (Colombia), 2018-2020.

The response to consumption for the interaction year and for corn material showed statistical differences between non-Bt and Bt corn (VT2P, VT3P). Values ranging from 4.7 to 98.8% corresponded to the 2019 collections that consumed non-Bt corn and the 2020 collections that consumed VT3P corn, respectively (**Table 5**, **Figure 3**).

**Table 5:** Means ± SD of mortality rate and growth inhibition 7 days after consuming (dac) non-Bt, VT2P and VT3P corn on *S. frugiperda* collected between 2018 and 2020 in HC.

Year	Corn material	No.	Mortality rate (%)^1a^	H&T^2b^	%GI^3c^
2018	Non-Bt	512	7.0 ± 3.0 b	0.0	0.0
2019	Non-Bt	768	4.7 ± 4.4 b	0.0	0.0
2020	Non-Bt	896	8.4 ± 1.5 b	0.0	0.0
2018	VT2P	2,048	97.6 ± 3.5 a	97.5 a	57.6 ± 8.0 b
2019	VT2P	3,072	95.9 ± 9.7 a	95.8 a	86.1 ± 8.7 a
2020	VT2P	3,584	98.3 ± 3.7 a	97.7 a	77.0 ± 18.6 ab
2018	VT3P	2,048	97.5 ± 3.8 a	97.3 a	61.9 ± 8.7 ab
2019	VT3P	3,072	98.6 ± 2.4 a	98.6 a	82.0 ± 4.8 ab
2020	VT3P	3,584	98.7 ± 2.9 a	98.3 a	75.0 ± 22.3 ab

n: Number of total larvae assessed each year.

SV-1035: conventional corn hybrid (expressing no Bt protein), control.

^1^Mortality: dead larvae + larvae and/or larvae weighing ≤10 mg), ± STDEV.

^3^Percentage of weight loss for live larvae 7 dac, with respect to the control (non-Bt corn).

In each column, values followed by different letters are statistically different (P<0.05) according to Tukey’s test and GLM for multiple comparisons.

^2^ Mortality rate: H&T corrected mortality.

a ANOVA results: df=8; F=462.57; P P<0.001.

b ANOVA results: df=8; F=756.52; P<0.001.

c ANOVA results: df=8; F=62.73; P<0.001.

**Figure 4:**
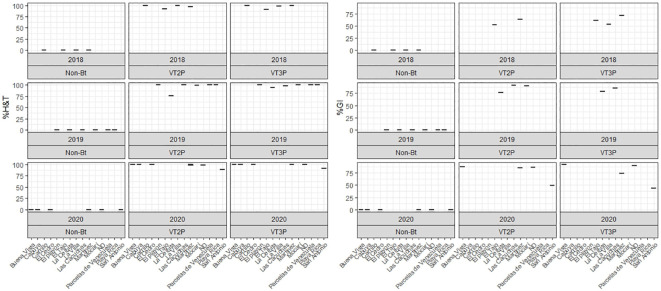
Response of *S. frugiperda* populations collected in the HC (2018-2020), according to % Mortality Rate, % H&T and Growth Inhibition (%GI), because of consuming of corn materials with VT2P and VT3P technology.

**Figure 5:**
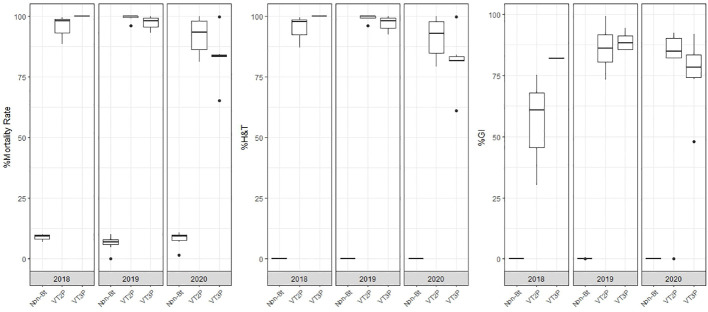
Response of *S. frugiperda* collected in GVMR (2018-2020), depending on the percentage of mortality rate, H&T and Growth Inhibition (%IG), due to consumption of VT2P and VT3P corn materials.

About collections made between 2018 and 2020, mortality due to consumption of VT2P and VT3P corn material was >95.0% (H&T), there were no statistical differences between materials (**Table 5**, **Figure 4**) and values ranged from 57.6 to 86.1% for the %GI parameter. The effect of consuming VT2P was statistically different for populations collected in 2018 compared to the rest (**Table 5**). The mortality rate (H&T) for the assessed populations was between 75.6 and 100.0%. The lowest mortality rate was for the population collected in La Ceibita (Cereté) in 2019, due to VT2P corn consumption (**Table 6**, **Figure 4**). Of the 16 populations that were fed VT3P, seven showed 100.0% mortality while the rest were between 89.4 and 99.6% (**Table 6**, **Figure 5**). The lowest growth inhibition percentage corresponded to larvae collected in 2020 in San Antonio (Cereté) and fed with VT3P corn (43.9%). In contrast, the highest value for %GI was for the population collected in Cabuya (San Carlos) in 2020 and fed with VT2P (**Table 6**, **Figure 4**).

**Table 6:** Mortality rate (%H&T) and growth inhibition (%) 7 days after consuming (dac) VT2P and VT3P corn on *S. frugiperda* populations collected between 2018 and 2020 in HC.

Year	Municipality	Population	VT2P		VT3P	
			% H&T^1^	%GI^1^	% H&T^1^	%GI^1^
2018	Cereté	El Tajo	92.3	51.9	91.5	60.9
2018	Cereté	Martinez	97.6	63.3	99.6	71.0
2018	San Benito de Abbad	La Villa	100.0	–	98.2	53.7
2018	San Pelayo	Carrillo	100.0	–	100.0	–
2019	Cereté	La Ceibita	75.6	76.0	94.0	78.6
2019	Cereté	Las Cacuchas	99.8	91.7	97.4	85.5
2019	Cereté	Playa Rica	100.0	–	100.0	–
2019	Lorica	El Playon	1000	–	100.0	–
2019	Monteria	Mocarí	99.2	90.5	100.0	–
2019	Tierra Alta	Parcelas de Venezuela	100.0	–	100.0	–
2020	Cereté	Buena Vista	99.8	87.0	99.6	91.9
2020	Cereté	El Cedro	100.0	–	100.0	–
2020	Cereté	Martinez	99.5	85.4	99.9	74.3
2020	Cereté	ND	98.7	86.4	99.6	90.2
2020	Cereté	San Antonio	86.4	49.1	89.4	43.9
2020	San Carlos	Cabuya	100.0	–	100.0	–

^1^Mortality rate: H&T corrected mortality.

^2^Percentage of weight loss for live larvae 7 dac, with respect to the control (non-Bt corn).

### Susceptibility of geographic valley of the Magdalena river populations

The populations assessed in GVMR from 2018 to 2020, showed mortality rates between 85.6 and 99.9% due to Bt corn consumption, which were statistically different from what had been seen in non-Bt corn (**Table 7**). With respect to mortality (H&T), values between 82.3 and 99.9% were recorded for *S. frugiperda* larvae fed with VT3P corn. The lowest value (82.3%) was recorded for populations collected in 2020 (**Table 7**). With reference to the %GI parameter, observations made in 2018 which were fed with VT2P corn and those made in 2019 fed with VT3P corn, were statistically different recording values of 55.4 and 89.0%, respectively (**Table 7**, **Figure 5**).

**Table 7:** Means ± SD of mortality rate and growth inhibition 7 days after consuming (dac) non-Bt, VT2P and VT3P corn on *S. frugiperda* collected from 2018 to 2020 in GVMR.

Year	Corn material	No.	Mortality rate (%)^1^	H&T^2^	%GI^3^
2018	Non-Bt	384	8.9 ± 1.6 c	–	–
2019	Non-Bt	896	6.4 ± 3.2 c	–	–
2020	Non-Bt	768	8.1 ± 3.4 c	–	–
2018	VT2P	1,536	95.2 ± 6.0 a	94.7 a	55.4 ± 22.9 a
2019	VT2P	3,584	99.2 ± 1.4 a	99.2 a	86.2 ± 10.9 a
2020	VT2P	3,072	91.8 ± 7.8 ab	91.0 ab	69.9 ± 39.3 a
2018	VT3P	1,536	99.9 ± 0.1 a	99.9 a	82.0 ± 0.0 a
2019	VT3P	3,584	97.2 ± 2.8 a	96.9 a	89.0 ± 3.9 a
020	VT3P	3,072	83.2 ± 10.9 b	81.5 b	75.8 ± 15.0 a

SV-1035: conventional corn hybrid (expressing no Bt protein), control.

^1^Mortality: dead larvae + larvae and/or larvae weighing ≤10 mg).

^2^Mortality rate: H&T corrected mortality.

^3^Percentage of weight loss for live larvae 7 dac, with respect to the control (non-Bt corn).

In each column, values followed by different letters are statistically different (P<0.05) according to Tukey’s test and GLM for multiple comparisons.

a ANOVA results: df=8; F=212.76; P<0.001.

b ANOVA results: df=8; F=235.69; P<0.001.

c ANOVA results: df=8; F=80.65; P<0.001.

**Figure 6:**
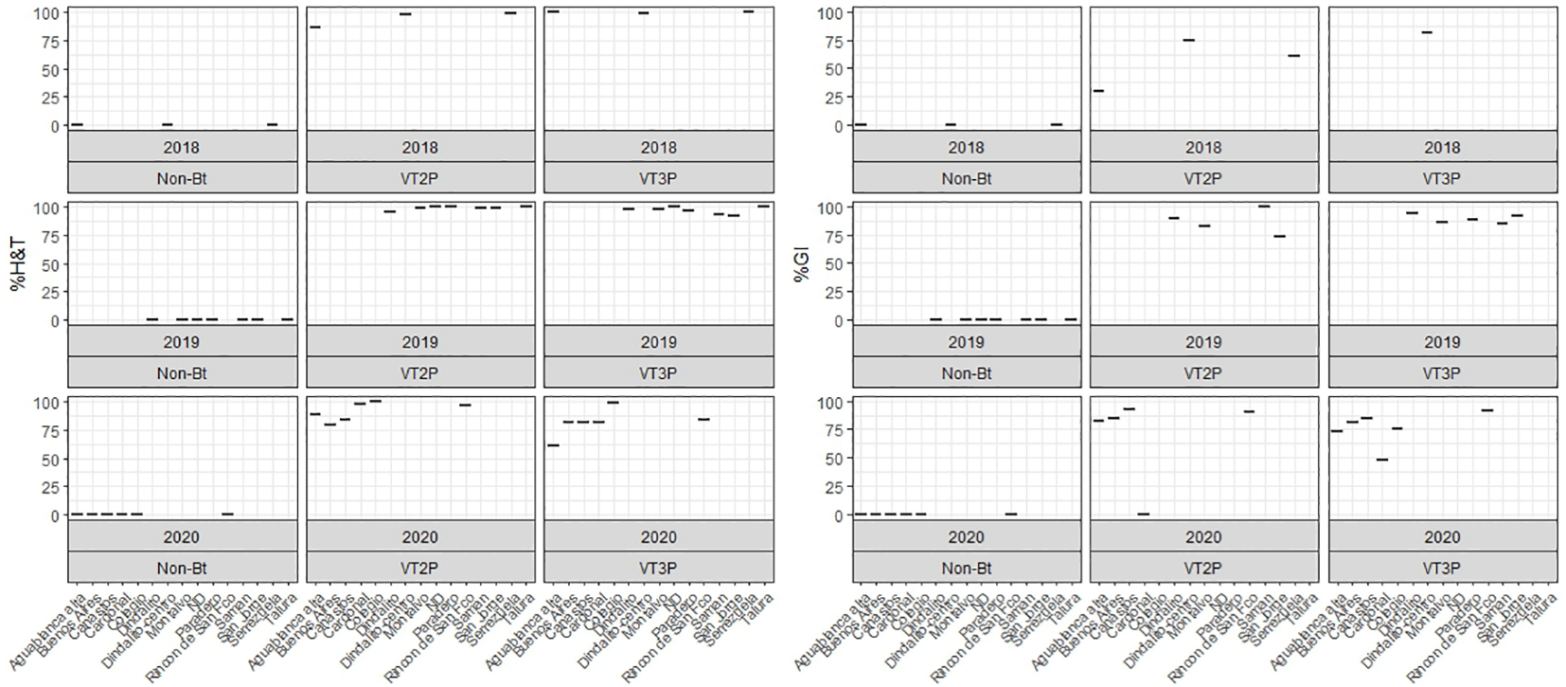
Response of *S. frugiperda* populations according to % Mortality Rate, % H&T and Growth Inhibition (%GI), due to consuming corn materials with VT2P and VT3P technology in GVMR (Colombia) 2018-2020.

The behavior of each of the 16 populations assessed in GVMR for mortality % (H&T) and fed with VT2P varied between 77.5 and 100%. The lowest value was recorded for the population collected in Buenos Aires (Ibagué, Tolima) in 2020, with growth inhibition of >80.0% (**Table 8**, **Figure 6**). In the case of larvae fed with VT3P corn, mortality values (H&T) were between 76.6 and 99.6%. Values for %GI between 48.1 and 94.4% were recorded for collections in Cardonal (Espinal, 2020) and Dindalito (Espinal, 2018) (**Table 8**, **Figure 6**).

**Table 8:** Mortality rate and growth inhibition 7 days after consuming (dac) non-Bt, VT2P and VT3P corn on *S. frugiperda* populations collected from 2018 to 2020 in GVMR.

Year	Municipality	Population	VT2P		VT3P	
			% H&T^1^	%GI^1^	% H&T^1^	%GI^1^
2018	Espinal	Aguablanca Alta	87.0	30.2	100.0	–
2018	Espinal	Dindalito-centro	97.7	75.1	99.8	82.0
2018	Guamo	Serrezuela	99.4	60.9	100.0	–
2019	Espinal	Dindalito	96.1	89.3	97.9	94.4
2019	Espinal	Montalvo	98.9	82.9	98.1	85.5
2019	Espinal	ND	100.0	–	100.0	–
2019	Espinal	Saman	99.8	99.4	93.1	85.3
2019	Espinal	Talura	100.0	–	100.0	–
2019	Flandes	Paradero	100.0	–	97.0	88.4
2019	Guamo	San Jorge	99.6	73.3	92.4	91.3
2020	Espinal	Aguablanca Alta	88.2	82.2	61.0	73.5
2020	Espinal	Canastos	83.6	92.4	81.5	84.3
2020	Espinal	Cardonal	97.8		81.7	48.1
2020	Espinal	Rincon de San Fco	97.2	90.2	83.9	92.0
2020	Flandes	Colegio	100.0	–	99.6	76.0
2020	Ibagué	Buenos Aires	79.1	84.8	81.5	80.8

^1^Mortality rate: H&T corrected mortality.

^2^Percentage of weight loss for live larvae 7 dac, with respect to the control.

### Susceptibility of Orinoquia populations

**Figure 7:**
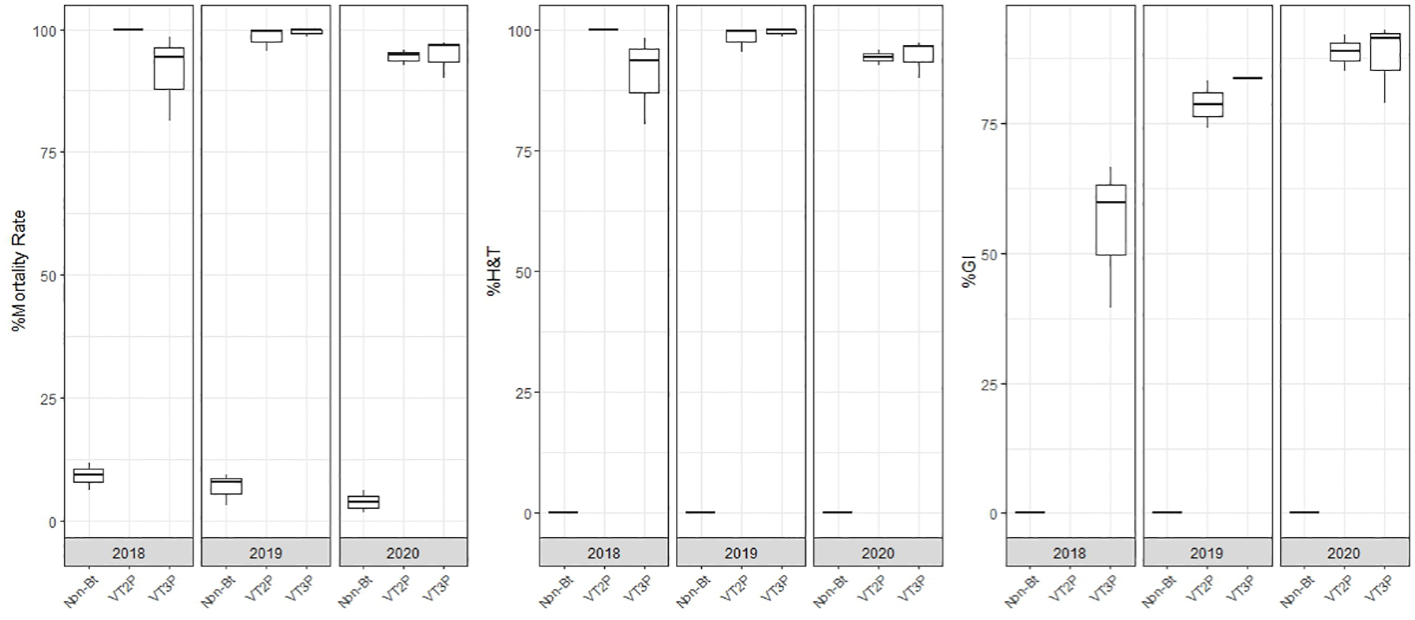
Response of *S. frugiperda* collected in Orinoquia (2018-2020), depending on the percentage of mortality rate, H&T and Growth Inhibition (%IG), due to consumption of VT2P and VT3P corn materials.

**Figure 8:**
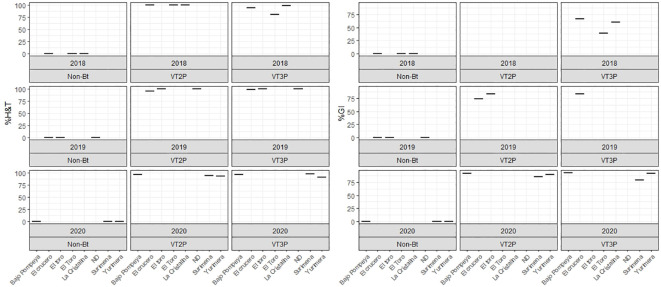
Response of *S. frugiperda* populations according to % Mortality Rate, % H&T and Growth Inhibition (%GI), due to consuming corn materials with VT2P and VT3P technology in Orinoquia (Colombia) 2018-2020.

For the collections made between 2018 and 2020 in the Orinoco, it was possible to establish values for the mortality rate parameter at between 3.9 and 100%, which showed significant differences because of consuming of non-Bt corn and Bt corn (VT2P and VT3P) (**Table 9**). There was a statistical difference in mortality (H&T) between the larvae collected in 2018 and fed with VT3P (90.7%), compared to those collected in 2018 and larvae fed with VT2P (100.0%) (**Table 9**, **Figure 7**). There were also statistical differences for growth inhibition, because of Bt corn consumption, with values ranging from between 55.3 and 88.7%. These corresponded to the 2018 collections which were fed with VT3P, and the larvae collected in 2020 fed with VT2P, respectively. Regarding the nine populations fed with VT2P corn, mortality rate values (H&T) ranged between 92.7 and 100.0%. Values for %GI were 78.8% for larvae collected in 2019 in El Crucero (Granada) and 92.1% for the collections in 2020 in Bajo Pompeya (Villavicencio) (**Table 9**). The mortality rate (H&T) values were higher than 80.0% because of consuming VT3P corn. The lowest mortality rate (H&T) was for the population collected in 2018 in Toro (Puerto López) (**Table 10**). In contrast, values for %GI ranged from 39.5 to 93.2%, with the lowest value given to the population collected in El Toro (Puerto Lopez) in 2018 (**Table 10**, **Figure 8**).

**Table 9:** Means ± SD of mortality rate and growth inhibition 7 days after consuming (dac) non-Bt, VT2P and VT3P corn, on *S. frugiperda* collected between 2018 and 2020 in the Orinoquia.

Year	Corn material	No.	Mortality rate (%)^1^	H&T^2^	%GI^3^
2018	Non-Bt	384	9.1 ± 2.7 c	–	–
2019	Non-Bt	384	6.8 ± 3.3 c	–	–
2020	Non-Bt	384	3.9 ± 2.3 c	–	–
2018	VT2P	1,536	100.0 ± 0.0 a	100.0 a	
2019	VT2P	1,536	98.4 ± 2.5 a	98.2 a	78.7 ± 6.4 a
2020	VT2P	1,536	94.5 ± 1.6 a	94.3 a	88.7 ± 3.5 a
2018	VT3P	1,536	91.4 ± 8.9 a	90.7 a	55.3 ± 14.1 b
2019	VT3P	1,536	99.5 ± 0.9 a	99.4 a	83.7 ± 0.0 a
2020	VT3P	1,536	94.7 ± 4.0 a	94.5 a	87.9 ± 7.8 a

SV-1035: conventional corn hybrid (expressing no Bt protein), control.

^1^Mortality: dead larvae + larvae and/or larvae weighing ≤10 mg).

^2^Mortality rate: H&T corrected mortality.

^3^Percentage of weight loss for live larvae 7 dac, with respect to the control non-Bt corn).

In each column, values followed by different letters are statistically different (P<0.05) according to Tukey’s test and GLM for multiple comparisons.

a ANOVA results: df=8; F=460.81; P<0.001.

b ANOVA results: df=8; F=701.16 P<0.001.

c ANOVA results: df=7; F=145.63; P<0.001.

**Table 10:** Mortality rate and growth inhibition 7 days after consuming (dac) non-Bt, VT2P and VT3P corn on *S. frugiperda* populations collected from 2018 to 2020 in the Orinoquia.

Year	Municipality	Population	VT2P		VT3P	
			% H&T^1^	%GI^1^	% H&T^1^	%GI^1^
2018	Granada	El Crucero	100.0	–	93.6	66.6
2018	Pto Gaitan	La Cristalina	100.0	–	98.3	59.8
2018	Pto Lopez	El Toro	100.0	–	80.2	39.5
2019	Granada	Crucero	95.1	74.2	98.3	83.7
2019	Pto Lopez	El Toro	99.6	83.2	100.0	–
2019	Pto Lopez	ND	100.0	–	100.0	–
2020	Puerto López	Yurimera	92.7	89.0	89.9	91.5
2020	San Carlos de Guaroa	Surimena	94.4	85.1	97.1	78.9
2020	Villavicencio	Bajo Pompeya	95.7	92.1	96.5	93.2

^1^Mortality rate: H&T corrected mortality.

^2^Percentage weight loss for live larvae 7 dac, with respect to the control (non-Bt corn).

### Susceptibility of GVCR populations

There were statistical differences for the mortality rate for the interaction year and corn material (**Table 11**). Mortality rate values ranged from 4.7 to 100.0% due to consuming Non-Bt and Bt corn (VT2P - VT3P), respectively (**Table 11**). All six populations collected in the GVCR showed 100% mortality in response to consuming plant material with VT2P and VT3P technologies (**Table 12**).

**Table 11:** Means ± SD of mortality rate and growth inhibition 7 days after consuming (dac) non-Bt, VT2P and VT3P corn on *S. frugiperda* collected between 2018 and 2020 in GVCR.

Year	Corn material	N	Mortality rate (%)^1a^	H&T^2^	%GI^3^
2018	Non-Bt	128	4.7 ± NA b	–	–
2019	Non-Bt	256	8.6 ± 3.3 b	–	–
2020	Non-Bt	512	6.6 ± 4.2 b	–	–
2018	VT2P	512	100.0 a	100.0 a	
2019	VT2P	2,048	100.0 a	100.0 a	–
2020	VT2P	2,048	100.0 a	100.0 a	–
2018	VT3P	512	100.0 a	100.0 a	
2019	VT3P	2,048	100.0 a	100.0 a	–
2020	VT3P	2,048	100.0 a	100.0 a	–

SV-1035: conventional corn hybrid (expressing no Bt protein), control.

^1^Mortality: dead larvae + larvae and/or larvae weighing ≤10 mg).

^2^Mortality rate: H&T corrected mortality.

^3^Percentage of weight loss for live larvae 7 dac, with respect to the control (non-Bt corn).

In each column, values followed by different letters are statistically different (P<0.05) according to Tukey’s test and GLM for multiple comparisons.

a ANOVA results: df=8; F=1,666.05; P<0.001.

**Table 12:** Mortality rate and growth inhibition 7 days after consuming (dac) non-Bt, VT2P and VT3P corn on *S. frugiperda* populations collected from 2018 to 2020 in GVCR.

Year	Municipality	Population	VT PRO^®^		VT3P	
			% H&T^1^	%GI^1^	% H&T^1^	%GI^1^
2018	Cartago	Guayabita	100.0	–	100.0	–
2019	Palmira	ND	100.0	–	100.0	–
2019	Roldanillo	Morelia	100.0	–	100.0	–
2020	Ginebra	La selva	100.0	–	100.0	–
2020	Riofrio	ND	100.0	–	100.0	–
2020	Roldanillo	Tierra Blanca	100.0	–	100.0	–
2020	Yotoco	LA Legua	100.0	–	100.0	–

^1^Mortality rate: H&T corrected mortality.

^2^Percentage of weight loss for live larvae 7 dac, with respect to the control (non-Bt corn).

NA, Not available.

## Discussion

The susceptibility of *S. frugiperda* to corn materials with *B. thuringiensis* insecticidal endotoxins can be influenced by multiple biotic and abiotic factors. However, the data reported in this study is about the susceptibility of *S. frugiperda* populations collected in the four agricultural subregions of Colombia after consuming VT2P (cry1A.105-Cry2Ab2) and VT3P (Cry1A.105-Cry2Ab2-Cry3Bb1) corn by mortality rate and percentage of growth inhibition. These results agree with the results reported under field conditions (53), about the effect of corn materials expressing Cry1A.105 and Cry2Ab2 proteins.

Previous studies on the effect of the consumption of Bt corn expressing Cry1A.105 and Cry2Ab2 proteins on the survival of *S. frugiperda* larvae indicated mortality rates from 97.0 to 100.0% (54–57). In this study, the mortality rate for *S. frugiperda* populations assessed from 2018 to 2020 in Colombia (because of consuming corn containing VT2P and VT3P technologies) confirms their susceptibility to these proteins which have produced an effective control for *S. frugiperda* under this study’s conditions. This contrasts with the results reported by (56) that showed 100% mortality 21 days after consuming corn containing Cry1A.105, Cry2Ab2, Cry3Bb1 proteins. Growth inhibition (GI) values greater than 57.0% denote a sublethal effect on *S. frugiperda* as a result of the consumption of VT2P and VT3P corn, generating an impact on the population over time, which with the proper implementation of refuge areas, will become a strategy that leads to the susceptibility of *S. frugiperda* over time (56, 58, 59).

Analyzing the impact of the consumption of Bt corn material on mortality and growth inhibition rates (according to the year collected and the corn material consumed), it was possible to determine the efficacy of Bt proteins on the populations of *S. frugiperda* assessed from 2018 to 2020 in Colombia. These results confirm the current susceptibility of *S. frugiperda* to VT2P and VT3P corn materials in the subregions assessed under the conditions in this study.

Assessing the impact of consuming VT2P and VT3P corn (according to four agricultural subregions of Colombia from which the *S. frugiperda* was collected), it was found that there was no statistical difference in mortality rates between the subregions, and that in regions such as the GVCR, all the populations that were assessed demonstrated high susceptibility to the proteins expressed in VT2P and VT3P corn. Additionally, larvae that managed to survive after seven days of consuming Bt corn, showed values for growth inhibition (%GI) that were higher than 74.0%, compared to larvae fed with non-Bt corn. It was confirmed that (regardless of origin), seven days of consuming corn with Cry1A.105, Cry2Ab2, Cry3Bb1 proteins produces a sublethal effect on live larvae according to their growth inhibition (56, 59).

In the case of the *S. frugiperda* populations collected in HC, the lowest mortality rates corresponded to the 2020 collections from San Antonio (Cereté) and coincided with the lowest percentages for growth inhibition (GI), after consuming VT2P and VT3P corn for seven days. This information establishes a baseline for future research aimed at detecting possible changes in the susceptibility of *S. frugiperda* in the HC subregion. (24).

In the case of GVMR, the lowest mortality rate value corresponded to the population collected in 2020 and fed with VT2P and VT3P corn. In contrast, the lowest growth inhibition percentage was recorded for larvae fed with VT2P corn in 2018 and for larvae fed with VT3P in 2020. This confirms the importance of *S. frugiperda* monitoring programs as a tool for identifying possible changes in susceptibility to commercially released proteins as early as possible (26, 28).

The mortality rate in Orinoquia between 2018 and 2020 shows the current susceptibility of *S. frugiperda* in that region. Moreover, the effect on larval development according to growth inhibition (with respect to larvae fed with Non-Bt corn) was sublethal (59).

The GVMR is the only region in Colombia where all *S. frugiperda* populations demonstrated high susceptibility consuming Bt corn expressing Cry1A.105 - Cry2Ab2 (VT2P) and Cry1A.105 - Cry2Ab2 - Cry3Bb1 (VT3P) proteins.

This information highlights the importance of constant monitoring (under field conditions) to detect variations in the susceptibility of *S. frugiperda* populations, and this is particularly important in tropical conditions. In addition, careful attention needs to be paid to the high genetic variability of *S. frugiperda* currently reported in Colombia (6, 30, 60). This monitoring also needs to be carried out to compare results obtained in laboratory conditions and the efficacy of technologies used in the field (26, 29).

Finally, these monitoring results should be treated with caution (the conditions of the study should be taken into consideration), when implementing strategies that encourage the susceptibility of *S. frugiperda* in Colombia.

## Data availability statement

The raw data supporting the conclusions of this article will be made available by the authors, without undue reservation.

## Author contributions

JR-C: Data curation, Formal analysis, Funding acquisition, Investigation, Methodology, Project administration, Supervision, Writing – original draft. SV: Investigation, Methodology, Supervision, Writing – review & editing. AR-C: Investigation, Methodology, Writing – review & editing. FS-G: Writing – review & editing, Resources. DR: Writing – review & editing, Supervision.
